# Linac-based cone-SRS for intracranial oligometastases: A comprehensive evaluation

**DOI:** 10.1097/MD.0000000000051372

**Published:** 2026-11-06

**Authors:** Menglin Yang, Rui Song, Jun Yang, Shengnan Dong, Tingting Qin, Yangyang Huang, Yibao Liu

**Affiliations:** a Department of Radiotherapy, The Second Affiliated Hospital of Zhengzhou University, Zhengzhou, Henan, China; b Department of Radiotherapy, The Third Affiliated Hospital of Zhengzhou University, Zhengzhou, Henan, China; c Department of Radiotherapy, Henan Province Hospital of TCM, Zhengzhou, Henan, China; d School of Nuclear Science and Engineering, East China University of Technology, Nanchang, Jiangxi, China.

**Keywords:** brain metastasis, comprehensive evaluation, cone, dosimetric parameters, LINAC, SRS, VMAT

## Abstract

**Background:**

This study aimed to determine whether linac-based cone plans offer a more comprehensive advantage over volumetric-modulated arc therapy (VMAT) plans in the stereotactic radiosurgery treatment of up to 5 intracranial oligometastases.

**Methods:**

For 59 lesions in 23 patients, noncoplanar multi-arc cone and noncoplanar multi-arc VMAT plans were designed and compared using dosimetry, plan complexity, delivery time, γ-passing rates, and radiobiological indices.

**Results:**

The cone plans had significant dosimetric and radiobiological advantages over the VMAT plans, showed no significant benefits in plan complexity and γ-passing rate, and a disadvantage in delivery time. The mean conformity index value for the cone plans was slightly smaller than that for the VMAT plans (0.64 vs 0.68, *P *> .05), whereas the cone plans had a much better mean gradient index value than that for the VMAT plans (1.79 vs 5.32, *P* < .001). The mean dose to normal brain tissue was 1.41 Gy for the cone plans and 1.61 Gy for the VMAT plans (*P *< .05). *V*_8 Gy_, *V*_5 Gy_, and *V*_3 Gy_ were significantly smaller for the cone plans than for the VMAT plans (all *P* < .05), except for V_12 Gy_, which showed no significance. As the number of lesions increased, the advantage of the smaller low-dose volume of the cone plans became more and more evident. Both plans obtained a mean tumor control probability of >99% for planning target volumes (*P* > .05), but the mean normal tissue complication probability of normal brain tissue was significantly lower for the cone plans than for the VMAT plans (*P *< .05), and both values were much <1%.

**Conclusion:**

The linac-based cone-stereotactic radiosurgery plans have more comprehensive advantages and are a better choice for up to 5 intracranial oligometastases.

## 1. Introduction

Intracranial metastases are the most common intracranial tumors in adults, with approximately 20% to 40% of cancer patients developing intracranial metastases during their illness.^[1]^ Intracranial oligometastases were previously defined as 1 to 4 metastases. Still, current study^[2]^ suggest that the number of lesions may be a minor prognostic factor when the total volume is limited. Stereotactic radiotherapy (SRT) for more than 4 metastases may also result in reasonable local control and survival results. Therefore, intracranial oligometastases are now defined as metastases that can be treated with SRT instead of whole brain radiotherapy with comparable or even better outcomes and with cognitive function preservation.^[3]^ In recent years, the role of single-fraction stereotactic radiosurgery (SRS) in the radiotherapy of intracranial oligometastases has become increasingly important.^[4]^ SRS causes minor neurocognitive damage to patients,^[5,6]^ improves patients’ overall survival,^[7,8]^ and is delivered in a short time (1 fraction), which is an important pattern in SRT radiotherapy nowadays.

In earlier years, SRS treatment of intracranial oligometastases was usually done on dedicated equipment such as the Gamma Knife.^[9–12]^ In recent years, the range of linac-based SRS applications has been expanding. This approach is more economical and cost-effective than Gamma Knife and CyberKnife, and it is more widely used in clinical practice.^[13–15]^ Brezovich et al^[16]^ concluded that SRS can be designed and delivered on a standard linac without needing a dedicated collimator system, with a spatial accuracy of better than 0.5 mm and dosimetric errors of <5%. A study by Thomas et al^[17]^ found that linac-based volumetric-modulated arc therapy (VMAT) plans had significant advantages over Gamma Knife plans in terms of plan quality, intermediate-dose reduction in normal brain tissue, and delivery time. Similar conclusions were made by Liu et al.,^[18]^ who compared linac-based and Gamma Knife-based SRS treatments and found that the linac-based VMAT plans had a smaller mean conformity index (CI) (1.19 vs 1.50, *P *< .001) and a higher mean gradient index (GI) (4.77 vs 3.65, *P *< .01). There was little difference between the 2 in the intermediate-dose volume (*V*_12 Gy_) (2.73 vs 3.06 cc, *P *= .58), but there was a 3- to 5-fold reduction in delivery time.

Although linac-based VMAT plans are commonly used for the SRS treatment of brain metastases, the large intermediate-dose volume (*V*_12 Gy_, known as the dose bridge) and low-dose volume (*V*_8 Gy_) of normal brain tissue caused by VMAT are unavoidable disadvantages, which can have a negative impact on organs at risk (OARs).^[19]^ Therefore, cone-SRS plans have a unique value in protecting OARs. Xu et al^[20]^ concluded that when cone- and VMAT-SRS treatment was carried out for arteriovenous malformations, the median *V*_12 Gy_ of normal brain tissue from the cone plans (2.4 vs 8.9 cc, *P* < .001) and median *V*_4 Gy_ (14.7 vs 58.5 cc, *P* < .001) were significantly reduced, which could greatly reduce the radionecrosis of normal brain tissue. Duan et al^[21]^ found that when SRS was used to treat a single, small brain metastasis, the cone plans were significantly more effective in terms of mean *V*_12 Gy_ (3.45 vs 10.97 cc, *P *< .05) and mean GI (2.66 vs 5.47, *P *< .05) than the VMAT plans.

However, the above studies have mainly focused on the vone-SRS treatment of single metastases; there are few reports on cone-SRS treatment cases of up to 5 intracranial oligometastases, which cannot provide the necessary theoretical support for physicians to recommend treatment. Therefore, it is essential to present the comprehensive comparison results between the cone-SRS and the VMAT-SRS plans of up to 5 intracranial oligometastases. In this study, the primary objective was to compare dosimetric and radiobiological differences between cone and VMAT techniques for stereotactic radiotherapy of patients with up to 5 intracranial oligometastases. The secondary endpoint was to evaluate clinically practical indicators, including plan complexity, delivery time, and γ-passing rates. Findings from this work aim to provide evidence-based references for clinical implementation of cone-SRS plans.

## 2. Materials and methods

### 2.1. Patient cohort

Twenty-three patients with intracranial oligometastases consecutively enrolled and treated with single-isocenter VMAT-SRS/fractionated stereotactic radiotherapy at our Department between January 2022 and January 2024 were retrospectively selected. Different plans were generated for the same cohort of patients to eliminate confounding interindividual variations in tumor and normal tissue characteristics, compensating for insufficient statistical power resulting from the relatively small sample size.

These patients had a total of 59 lesions with a maximum diameter of <15 mm, with a median of 2 lesions and a maximum of 5 lesions. Patient inclusion criteria included a precise pathological diagnosis of the primary tumor and magnetic resonance imaging enhancement scans confirming the presence of intracranial metastatic lesions. Patient exclusion criteria included age >80 years, severe underlying disease, a history of previous intracranial radiotherapy, poor general condition, and a Karnofsky performance status <70. The gross tumor volume (GTV) and planning target volume (PTV) were 0.30 to 1.26 cc (median 0.49 cc) and 0.68 to 2.97 cc (median 1.14 cc), respectively. The general conditions of the patients are shown in Table 1. This study was approved by the Ethics Committee of our hospital (no. 2023202). Written informed consent was obtained from the patients to use their anonymized data for the study.

**Table 1:** Summary of patients’ general conditions.

Parameters	Values
Age	51–79 (median 63)
Sex	15 males and 8 females
Primary location	Lung 14, breast 6, cervix 2, and kidney 1
Number of lesions	7 patients with 1 lesion, 5 patients with 2 lesions, 5 patients with 3 lesions, 3 patients with 4 lesions, and 3 patients with 5 lesions
Lesion location	Frontal lobe 31%, parietal lobe 17%, temporal lobe 24%, cerebellum 12%, and occipital lobe 16%
Prescription	20 Gy/1 fraction
GTV	Diameter 2.2–11.8 mm (median 5.6 mm) and volume 0.30–1.26 cc (median 0.49 cc)
PTV	Diameter 2.8–14.7 mm (median 7.1 mm) and volume 0.68–2.97 cc (median 1.14 cc)

GTV = gross tumor volume, PTV = planning target volume.

### 2.2. Positioning and contouring

Each patient underwent a spiral scan on a 16-row big-bore computed tomography (CT) scanner (Philips Medical Systems). CT scan parameters included 100 kV, 200 mA, 512 × 512 pixels, and a slice thickness of 1.0 mm. All patients were placed in a supine position with arms naturally straight and placed on both sides of the body. The head and neck were fixed using a thermoplastic mask (Guangzhou Klarity Medical Equipment Co., Ltd.). The CT scan range included the entire brain, and an extension of the tumor region of 15 cm superiorly and inferiorly for noncoplanar treatment.^[22]^

One to 2 mm slice-thick enhancement magnetic resonance imaging was fused with the patient’s CT. All GTVs were delineated on gadolinium-enhanced T1-weighted MR images. A senior medical physicist with >5 years of clinical experience in cranial radiotherapy contoured all GTVs and OARs, strictly following the brain metastasis contouring criteria stipulated by RTOG 9508, RTOG 0320, and RTOG 1119. All contoured structures subsequently received secondary peer review by a senior attending physician leading the treatment team. Any inter-evaluator discrepancies were resolved through collective discussion to achieve consensus prior to final GTV definition. The PTVs were created by adding an isotropic 1 mm margin to the GTVs.^[23]^ OARs to be delineated included normal brain tissue that did not exclude the GTVs, brainstem, optical pathways, lens, and bilateral hippocampus.^[24]^ For lesions adjacent to critical structures, including the brainstem and optic way, GTV margins were confined exclusively within the contrast-enhanced tumor region without extension into intact functional brain parenchyma.

### 2.3. Planning

To facilitate comparison, the prescribed dose was set uniformly at 20 Gy/1 fraction for both plans.^[25]^ To reduce the intermediate-dose bridge between lesions, all the redesigned cone plans and VMAT plans used noncoplanar multiple arcs.^[26]^ The cone plans had 1 treatment center per lesion, whereas the VMAT plans used a single-center model regardless of the number of lesions. The treatment centers of all VMAT plans were located at the geometric centers of the PTVs. In accordance with institutional planning protocols, arc lengths, starting angles, and cost functions were predefined for both cone and VMAT plans. The Monaco treatment planning system restricts cone plans to a maximum of 1000 monitor units (MUs) per arc. Given the prescribed single-fraction SRS dose of 20 Gy, cone plans necessitate multiple arcs; a 5‑arc configuration was selected in our cohort to balance mechanical collision avoidance, OAR sparing and delivery efficiency while guaranteeing robust plan quality. Specifically, all cone plans consisted of 5 140° arcs delivered at table angles of ±10°, ±45°, and 90°. By contrast, conventional non‑coplanar single‑isocenter VMAT plans comprised four 180° arcs at table angles of ±45° and ±90°, +1 full 360° arc at 0° table angle. Arc start and stop angles were adjusted as needed to prevent gantry‑table collision.

To balance calculation efficiency and dosimetric accuracy, the dose grid was set to 1.0 mm, and statistical uncertainty was limited to 0.5% per control point, with a 0 to 1 mm multi-leaf collimator (MLC) margin. All plans were normalized to a 100% prescription dose covering 98% of the PTVs and a 100% prescription dose covering >99% of the GTVs, and it was ensured that all hotspots (between 120% and 150%) were located within the GTVs. If OARs overlapped the PTVs, the minimum dose for the PTVs was at least 70% of the prescribed dose.

To minimize bias, all plans were designed by a single senior medical physicist, while dosimetric data collection was performed independently by another investigator blinded to plan allocation. All plans were recalculated in Monaco treatment planning system (V5.4, Elekta Solution AB, Kungstensgatan 18) using the Monte Carlo algorithm.^[27]^ All plans were delivered by an Infinity accelerator equipped with Aktina cone collimators (5, 7, 10, 12, and 15 mm) and Agility collimators (5 mm MLC, 80 pairs). The cone diameter was selected based on target coverage, and accurate switching of collimators was ensured. All plans were delivered using the 6 MV flattening filter-free beam (maximum dose rate 1400 MU/min).^[28,29]^

### 2.4. Dosimetric evaluation

Dosimetric evaluation of the cone and VMAT plans was performed in terms of minimum dose *D*_98%_, maximum dose *D*_2%_, mean dose *D*_mean_, CI, GI of PTV, and maximum dose or dose volume of OARs.

CI = TV2 PIV/(TV × PIV), where TV_PIV_ is the volume of the PTV surrounded by 100% of the prescription dose, whereas TV is the volume of the PTV and PIV is the volume surrounded by 100% of the prescription dose; a CI approaching unity indicates improving conformity of the 100% prescription dose to the PTV.^[30]^ GI = PIV_50%_/PIV, where PIV_50%_ is the volume surrounded by 50% of the prescribed dose, and the smaller the GI, the better.^[30]^

All plans must meet dose limits for the brainstem, the optical pathway (optic nerve and optic chiasm), the lens, and the bilateral hippocampus.^[22,31–33]^ In particular, *V*_10 Gy_ < 0.5 cc and *D*_max_ < 15 Gy for the brainstem; *V*_8 Gy_ < 0.2 cc and *D*_max_ < 10 Gy for the optical pathway; *D*_max_ < 2 Gy for the lens; and *D*_max_ < 10 Gy for the bilateral hippocampus, respectively. The mean dose to normal brain tissue (*D*_mean_) and the volumes receiving doses above 12, 8, 5, and 3 Gy (*V*_12 Gy_, *V*_8 Gy_, *V*_5 Gy_, and *V*_3 Gy_) were also analyzed separately.^[34–36]^

### 2.5. Plan complexity, delivery time, and γ-passing rates

Plan complexity is evaluated based on MUs, which positively correlate with plan complexity.^[37]^

The delivery time of the cone plans includes the patients’ setup time per lesion, cone beam computed tomography (CBCT) position verification time per lesion, and beam-on time per lesion. We performed CBCT position verification for each lesion to reduce setup errors when switching the Cone plans in patients with multiple lesions.^[38]^ The delivery time for the VMAT plans consists of single setup time, single CBCT position verification time, and single beam-on time because there was only 1 treatment center. Delivery time for both plans was recorded when γ-passing rates were measured.

The γ-passing rates were measured using Sun Nuclear SRS MapCHECK, which was equipped with the StereoPHAN phantom (Sun Nuclear). Based on the American Association of Physicists in Medicine TG-218 report and other documentation,^[39,40]^ and taking into account the actual situation in the department, the acquisition parameters were set as 2%/2 mm > 95%, 2%/1 mm > 85%, and 1%/2 mm > 90%, respectively, while excluding data below 5% of the maximum dose.

### 2.6. Radiobiological indices

To evaluate the radiobiological effects of both plans, the tumor control probability (TCP) for PTVs and normal tissue complication probability (NTCP) for normal brain tissues were quantitatively calculated using an in-house procedure. The parameters below were derived from clinical data of patients receiving intracranial radiotherapy and are provided for reference only.

The expression for the calculation of TCP is shown below:


$$\begin{array}{c} TCP=1/(1+\left(\frac{TCD_{50}}{EUD}{)}^{4\gamma_{50}}\right) \end{array}$$ (1)

TCD_50_ in Equation (1) is the dose to control 50% of the tumor when irradiated uniformly, and γ_50_ is the expected change in TCP due to a 1% change in TCD_50_ dose. The brain metastases parameters TCD_50_, γ_50_, and α refer to Okunieff et al^[41]^ with values of 51.77 Gy, 2.28, and −13, respectively, whereas the equivalent uniform dose values were calculated using the model proposed by Gay et al.^[42]^


$$\begin{array}{c} EUD=\left(\underset{i=1}{\sum }\;(v_{i}D_{EQD_{i}}^{\alpha }\right){)}^{{\;}^{1}/{\;}_{\alpha }} \end{array}$$ (2)

*v*_*i*_ and DEQD_*i*_ represent a specific volume, *v*_*i*_, and its irradiation by a dose of DEQD_*i*_, respectively. α describes a unitless parametric factor of the dose volume to the tumors. To describe the single-fraction treatment model, DEQD_*i*_ in Equation (2) is the bioequivalent physical dose of 2 Gy/f, defined as follows:


$$\begin{array}{c} D_{{}_{EQD_{i}}}=D_{i}\left(\frac{\alpha /\beta +d}{\alpha /\beta +2}\right) \end{array}$$ (3)

The α/β in Equation (3) is the specific tumor’s linear-quadratic parameter. For brain lesions, the α/β ratio for tumor cells is usually 10 Gy,^[43]^ whereas that for normal brain tissue is usually 2 Gy.^[44]^ *D*_*i*_ represents that a given volume, *v*_*i*_, is irradiated with a dose of *D*_*i*_, and *d* represents a single dose.

The NTCP for normal brain tissue was calculated based on the Lyman–Kutcher–Burman model^[45–47]^ with the following expression:


$$\begin{array}{c} NTCP=\frac{1}{\sqrt{2\pi }}\int_{-\infty }^{t}e^{-\frac{t^{2}}{2}}dt \end{array}$$ (4)

$$\begin{array}{c} Among\;which\;t=\frac{D_{ef\;f}-TD_{50}}{mTD_{50}} \end{array}$$ (5)

Furthermore, *D*_eff_ in Equation (5) is calculated as:


$$D_{ef\;f}={\left(\underset{i=1}{\sum }\;v_{i}\overset{1/n}{\underset{EQD_{i}}{D}}\right)}^{n}$$ (6)

*D*_eff_ refers to the dose of NTCP that would result in a dose equal to the actual nonuniform irradiation if the whole organ were irradiated uniformly; TD_50_ is the dose that would result in a 50% probability of complication if the entire organ were irradiated uniformly; *m* is the slope of NTCP versus dose; *n* is the volume dependence at the organ; and *d* (*D*_*i*_, *v*_*i*_) are the bins of a differential dose volume histogram.^[47]^ DEQD_*i*_ is calculated as shown in Equation (3). According to Burman et al,^[47]^ the TD_50_, *m*, and *n* values for normal brain tissue are 60 Gy, 0.15, and 0.25, respectively.

### 2.7. Statistical analysis

Prior to statistical analysis, the Shapiro–Wilk test was adopted to examine the normality of all dosimetric and radiobiological variables. Parameters conforming to normal distribution were compared by paired-sample *t* test between cone and VMAT plans, while non-normally distributed data were analyzed using the Wilcoxon signed-rank test. Bonferroni correction was applied to adjust the significance level for multiple comparisons to avoid inflated type I error. All measured indexes were presented as mean ± standard deviation. After Bonferroni correction for multiple comparisons, differences with corrected *P *< .05 were considered statistically significant. Statistical analyses were performed with SPSS 26.0 (IBM Corporation).

## 3. Results

Both plans can produce qualified SRS plans. Table 2 summarizes the dosimetric parameters of the cone and VMAT plans in detail. All plans normalize to 100% of the prescribed dose, covering 98% of the PTVs; at this point, 100% of the prescribed dose covers 100% of the GTVs.

**Table 2:** Summary of dosimetric parameters for the cone versus VMAT plans (mean ± standard deviation).

Dosimetric parameters		Cone	VMAT	*P*
PTV	*D*_98%_ (Gy)	19. 63 ± 0.44	19.48 ± 1.06	.754
	*D*_2%_ (Gy)	30.69 ± 1.55	31.40 ± 2.35	.040*
	*D*_mean_ (Gy)	25.96 ± 0.80	26.47 ± 1.52	.025*
	CI	0.64 ± 0.09	0.68 ± 0.08	.130
	GI	1.79 ± 0.15	5.32 ± 1.51	<.001*
Brainstem	*V*_10 Gy_ < 0.5 cc	N/A	N/A	N/A
	*D*_max_ < 15 Gy	2.43 ± 1.53	3.97 ± 3.20	.044*
Optical pathway	*V*_8 Gy_ < 0.2 cc	N/A	N/A	N/A
	*D*_max_ < 10 Gy	1.34 ± 1.06	2.42 ± 1.88	.024*
Left lens	*D*_max_ < 2 Gy	0.50 ± 0.50	1.01 ± 0.81	.038*
Right Lens	*D*_max_ < 2 Gy	0.39 ± 0.33	0.83 ± 0.67	.121
Left hippocampus	*D*_max_ < 10 Gy	2.26 ± 2.22	3.71 ± 2.92	.012*
Right hippocampus	*D*_max_ < 10 Gy	2.45 ± 2.94	4.03 ± 4.37	.018*
Normal brain tissue	*D*_mean_ (Gy)	1.41 ± 0.84	1.61 ± 0.85	.030*
	*V*_12 Gy_ (cc)	12.30 ± 9.37	12.84 ± 8.81	.227
	*V*_8 Gy_ (cc)	24.26 ± 20.85	29.94 ± 25.31	.017*
	*V*_5 Gy_ (cc)	53.45 ± 50.26	71.57 ± 61.53	.006*
	*V*_3 Gy_ (cc)	116.02 ± 109.56	157.42 ± 121.58	.009*

Normal brain tissue refers to brain tissue from which GTV is not excluded.

CI = conformity index, GI = gradient index, GTV = gross tumor volume, N/A = not applicable, PTV = planning target volume, VMAT = volumetric-modulated arc therapy.

* After normality testing, paired-samples *t* tests were applied, and corrected values with a *P* < .05 are labeled; a ‘N/A’ indicates that all values are 0.

The cone plans were better than the VMAT plans for almost all PTV parameters. There was no significant difference in the *D*_98%_ between the cone and VMAT plans, whereas the mean value of *D*_2%_ was significantly smaller for the cone plans than for the VMAT plans (30.69 vs 31.40 Gy, *P *< .05). Similarly, the mean value of PTV *D*_mean_ for the cone plans was significantly smaller than that of the VMAT plans (25.96 vs 26.47 Gy, *P* < .05). The mean value of CI for the cone plans was slightly smaller than that of the VMAT plans (0.64 vs 0.68, *P* > .05), but the difference was not significant, whereas the mean value of GI for the cone plans was much better than that of the VMAT plans (1.79 vs 5.32, *P *< .001).

The cplans were better than the VMAT plans in almost all OAR parameters. The brainstem *D*_max_ and optical pathway *D*_max_ of the cone plans were significantly smaller than those of the VMAT plans (all *P* < .05) and were much smaller than the corresponding maximum dose limits of 15 and 10 Gy. The cone plans also favored the *D*_max_ of the lens, with the left lens being particularly significant (*P* < .05). Both plans protected the bilateral hippocampus well, but the *D*_max_ was significantly lower in the bilateral hippocampus for the cone plans than for the VMAT plans (all *P* < .05). The *D*_mean_ of normal brain tissue was 1.41 Gy for the cone plans and 1.61 Gy for the VMAT plans (*P* < .05). *V*_8 Gy_, *V*_5 Gy_, and *V*_3 Gy_ were significantly smaller for the cone plans than for the VMAT plans (all *P* < .05), except for *V*_12 Gy_, which showed no significance. The advantage of the cone plans increased as the evaluation dose of normal brain tissue decreased. For example, the decreased low-dose volume of normal brain tissue for the cone plans compared to the VMAT plans ranged from 18.97% for *V*_8 Gy_ to 26.30% for *V*_3 Gy_. Some OAR evaluation parameters were shown as not applicable (*V*_10 Gy_ for the brainstem and *V*_8 Gy_ for the optical pathway), as all had a 0 value.

To study the effect of different numbers of lesions on normal brain tissue, the number of lesions was divided into 2 groups, 1 to 2 and 3 to 5, as shown in Table 3. In plans with 1 to 2 or 3 to 5 lesions, there was little difference in the *D*_mean_ and *V*_12 Gy_ to normal brain tissue between both plans (all *P *> .05). However, the low-dose volumes of normal brain tissue (*V*_8 Gy_, *V*_5 Gy_, and *V*_3 Gy_) for the Cone plans at 1 to 2 lesions and 3 to 5 lesions were significantly smaller than those for the corresponding VMAT plans (all *P* < .05). Meanwhile, as the number of lesions increased, the advantage of the small low-dose volume of the cone plans became increasingly apparent. For example, at *V*_5 Gy_, the mean volume reduction of Cone plans for 1 to 2 lesions was 10.49 cc, and the mean volume reduction of cone plans for 3 to 5 lesions was 37.66 cc. This pattern also held under other normal brain tissue evaluation parameters (*D*_mean_, *V*_12 Gy_, *V*_8 Gy_, and *V*_3 Gy_). Figures 1–3 show the dose distributions of 3 representative patients, and it can be visualized that the low-dose volume of the cone plans is smaller than that of the corresponding VMAT plans; the advantage of the smaller low-dose volume of the cone plans (fewer intermediate-dose bridges in patients with multiple lesions) is shown more and more clearly with the increase in the number of lesions.

**Table 3:** Comparison of dosimetric parameters in normal brain tissue with different numbers of lesions (mean ± standard deviation).

Dosimetric parameters		1–2 lesions per patient			3–5 lesions per patient		
		Cone	VMAT	*P*	Cone	VMAT	*P*
Normal brain tissue	*D*_mean_ (Gy)	0.80 ± 0.17	1.03 ± 0.32	0.159	2.15 ± 0.64	2.34 ± 0.56	.131
	*V*_12 Gy_ (cc)	5.77 ± 2.10	6.67 ± 2.33	.074	20.45 ± 8.27	20.54 ± 7.56	.926
	*V*_8 Gy_ (cc)	10.06 ± 3.87	13.01 ± 5.01	.021*	42.01 ± 19.58	51.11 ± 20.48	.047*
	*V*_5 Gy_ (cc)	19.47 ± 7.93	29.96 ± 9.08	.035*	85.93 ± 48.20	123.59 ± 28.33	.031*
	*V*_3 Gy_ (cc)	42.61 ± 20.18	77.09 ± 18.13	.018*	187.77 ± 96.10	257.83 ± 91.98	.016*

Normal brain tissue refers to brain tissue from which GTV is not excluded.

VMAT = volumetric-modulated arc therapy.

* After normality testing, paired-samples *t* tests were applied, and corrected values with a *P* < .05 are labeled.

**Figure 1.**
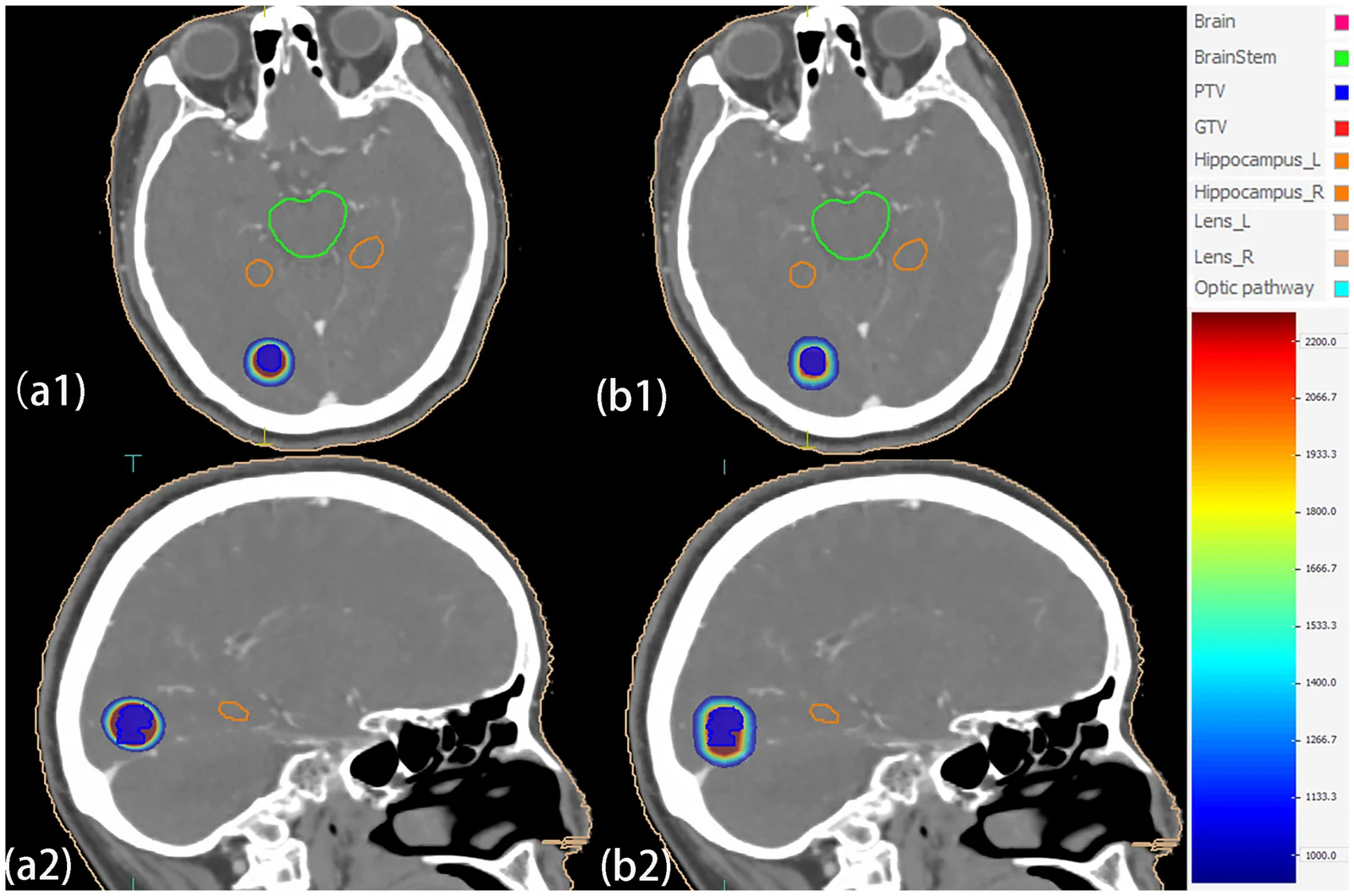
Comparison of dose distributions between the cone plan (a1 and a2) and the VMAT plan (b1 and b2) when the lesion number is 1. The dose rendering scale is from 1000 to 2100 cGy, with the top row being a transverse plane and the bottom row being a sagittal plane. The blue structure is the PTV, the green structure is the brainstem, and the yellow structure is the bilateral hippocampus. GTV = gross tumor volume, PTV = planning target volume, VMAT = volumetric-modulated arc therapy.

**Figure 2.**
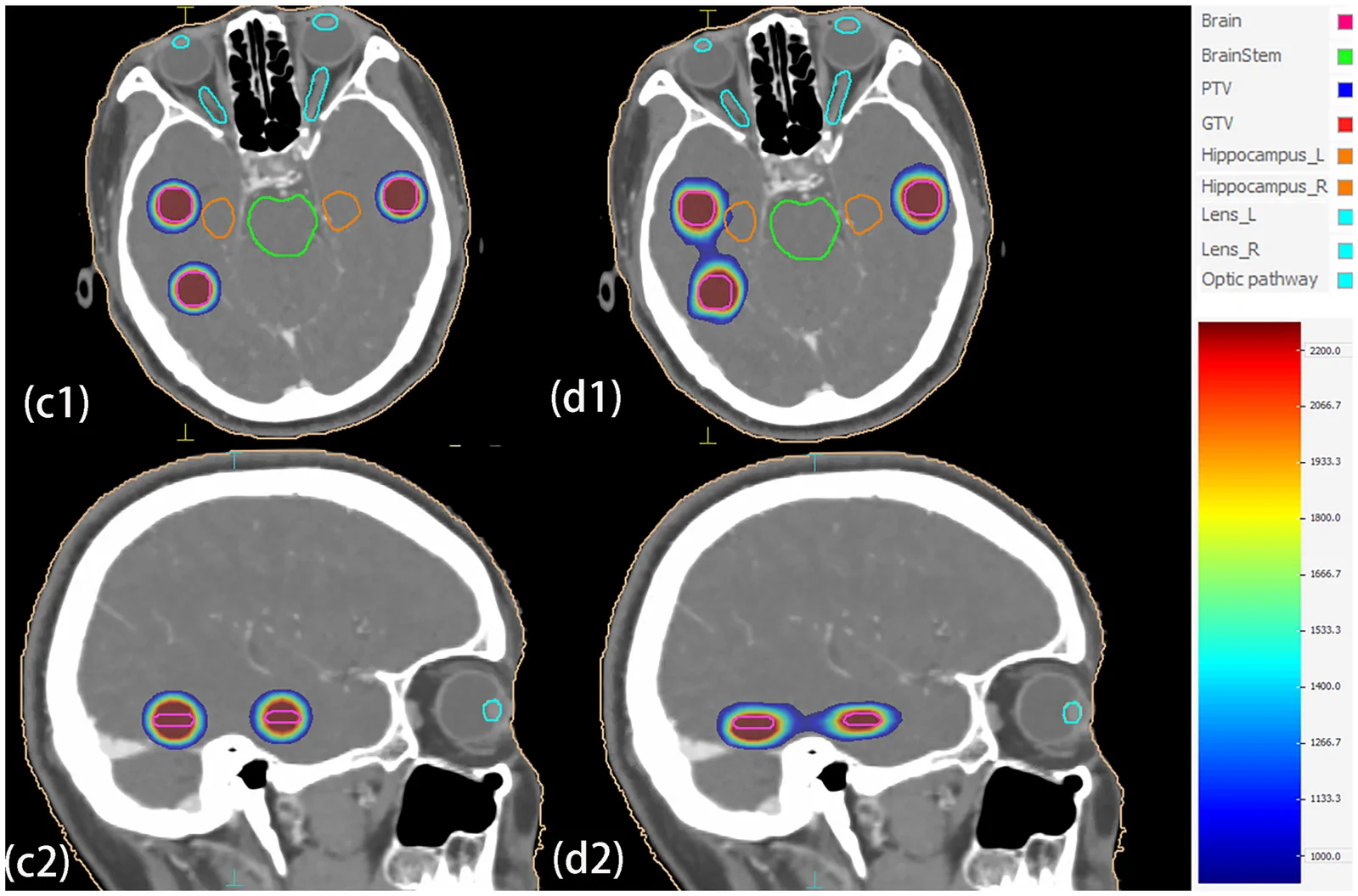
Comparison of dose distributions between the cone plan (c1 and c2) and the VMAT plan (d1 and d2) when the lesion number is 3. The dose rendering scale is from 1000 to 2100 cGy, with the top row being a transverse plane and the bottom row being a sagittal plane. The pink structure is the PTV, the green structure is the brainstem, the yellow structure is the bilateral hippocampus, and the cyan structure is the optic pathway. GTV = gross tumor volume, PTV = planning target volume, VMAT = volumetric-modulated arc therapy.

**Figure 3.**
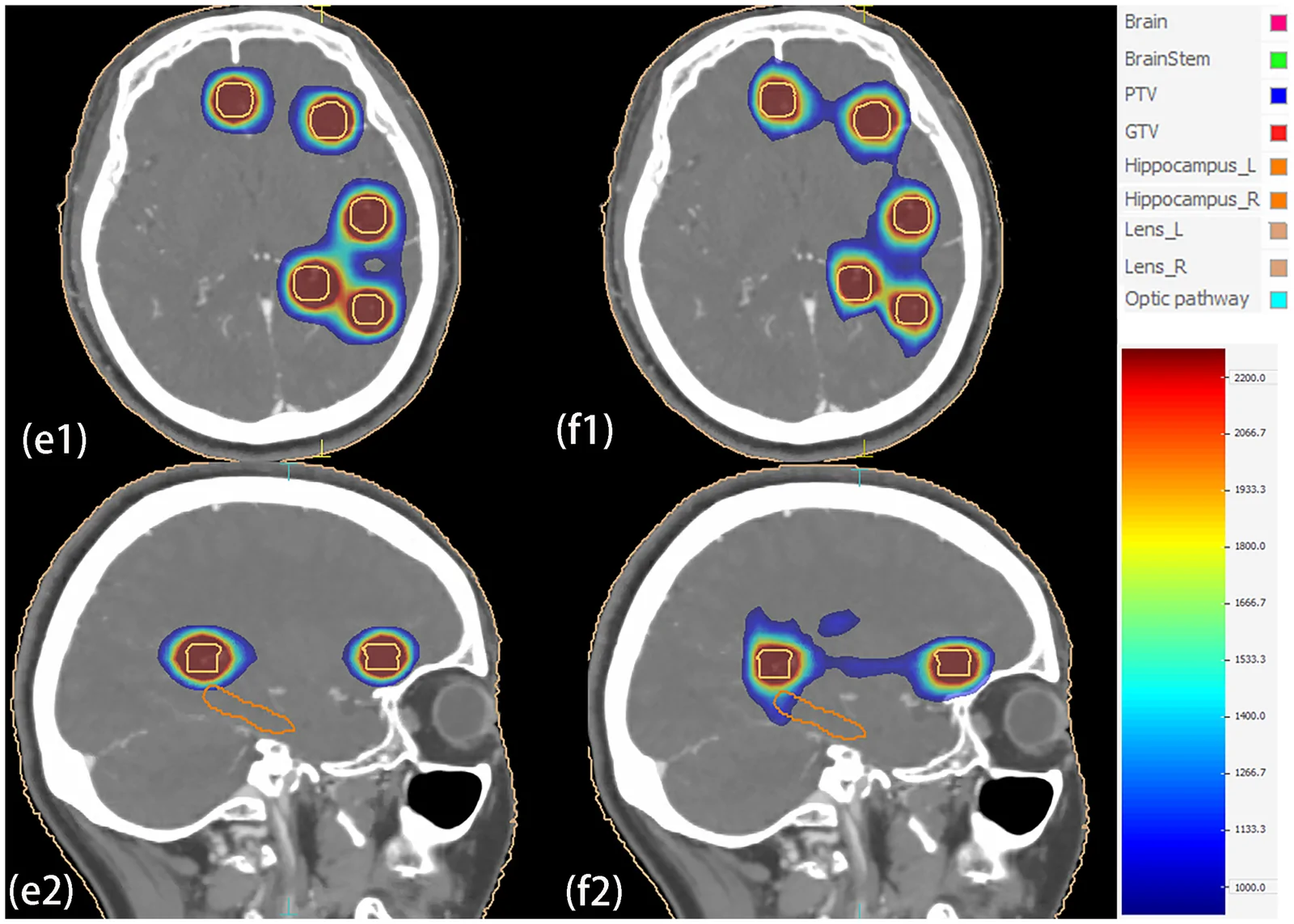
Comparison of dose distributions between the cone plan (e1 and e2) and VMAT plan (f1 and f2) when the lesion number is 5. The dose rendering scale is from 1000 to 2100 cGy, with the top row being a transverse plane and the bottom row being a sagittal plane. The yellow structure is the PTV, and the yellow structure is the bilateral hippocampus. GTV = gross tumor volume, PTV = planning target volume, VMAT = volumetric-modulated arc therapy.

As shown in Table 4, the MUs of the cone plans did not differ significantly (*P* > .05) from the VMAT plans. The differences between the γ-passing rates of both plans were insignificant (all *P* > .05) under the 3 acquisition parameters, and all met the requirements. Unsurprisingly, the delivery time for the cone plans was significantly longer than the VMAT plans (*P* < .05).

**Table 4:** Summary of plan parameters for the cone and VMAT plans (mean ± standard deviation).

Plan parameters		Cone	VMAT	*P*
MUs	N/A	10,066.23 ± 5796.63	9997.21 ± 3521.38	.938
γ-passing rate (%)	2%/2 mm	99.19 ± 0.28	99.01 ± 0.32	.435
	1%/2 mm	97.82 ± 1.32	97.24 ± 0.91	.113
	2%/1 mm	94.41 ± 2.20	94.55 ± 2.87	.218
Delivery time (s)	N/A	1401.67 ± 841.65	647.07 ± 159.62	.011*

MUs = monitor units, N/A = not applicable, VMAT = volumetric-modulated arc therapy.

* After normality testing, paired-samples *t* tests were applied, and corrected values with a *P* < .05 are labeled.

Table 5 shows the TCP for PTV and NTCP for normal brain tissue. Both plans obtained an average TCP of >99% for PTV (*P *> .05). The mean value of NTCP of normal brain tissue for the Cone plans was significantly lower than that for the VMAT plans (*P* < .05), with mean values of 1.08 × 10^−7^% and 3.79 × 10^−7^%, respectively, which were both much <1%.

**Table 5:** Summary of radiobiological indices for cone and VMAT plans (mean ± standard deviation).

Radiobiological indices	Cone	VMAT	*P*
PTV TCP (%)	99.13 ± 0.21	99.34 ± 0.38	.351
Normal brain tissue NTCP (× 10^−7^%)	1.08 ± 0.93	3.79 ± 1.35	.001*

NTCP = normal tissue complication probability, TCP = tumor control probability, VMAT = volumetric-modulated arc therapy.

* After normality testing, paired-samples *t* tests were applied, and corrected values with a *P* < .05 are labeled.

## 4. Discussion

This study comprehensively compared the dosimetry, plan complexity, delivery time, γ-passing rates, and radiobiological indices of the cone and VMAT plans in 23 patients with up to 5 intracranial oligometastases. The results showed that the cone plans were superior in almost all dosimetric parameters of PTVs and OARs. In contrast, the cone plans did not differ significantly from the VMAT plans regarding MUs and γ-passing rates. Compared to the dosimetric advantages, the delivery time of the cone plans can be longer, causing some disturbances to the patients’ treatment. The mean TCP of the PTVs was >99% for both plans, whereas the mean NTCP of normal brain tissues was much <1% for both plans, and the mean NTCP of normal brain tissues for the cone plans was significantly smaller than that for the VMAT plans. In conclusion, the cone-SRS plans have a comprehensive advantage for patients with up to 5 intracranial oligometastases.

Table 2 shows that the cone plans were better than the VMAT plans in almost all PTV parameters. For example, the mean *D*_2%_ was significantly smaller for the cone than for the VMAT plans (30.69 vs 31.40 Gy, *P *< .05). Similarly, the *D*_mean_ for the cone plans was significantly smaller than that for the VMAT plans (*P* < .05). The above results may be related to the cone plans’ lack of intensity modulation capability.^[48]^ Because the cone plans’ apertures are circular spots, unlike the VMAT plans, where different segments overlap within the PTVs; thus, the PTV dose is more homogeneous for the cone plans. The mean CI value for the cone plans was slightly smaller than that for the VMAT plans (0.64 vs 0.68, *P *> .05), but the difference is insignificant. This result is also associated with the cone plans’ lack of intensity modulation capability.^[48]^ The cone plans lack continuous apertures, and only several discrete apertures can be selected. Whereas some patients’ PTVs are not of standard spherical shape,^[49]^ this results in the irradiated volume of the cone plans being determined by the cone size. When the patients’ PTVs are smaller in volume and larger in diameter, a lower CI value occurs, which can be seen visually from the PTVs versus dose distribution relationship in the sagittal planes of Figures 1–3.

The mean values of GI for cone plans and VMAT plans were 1.79 and 5.32, respectively (*P *< .001). Compared to MLC, the cone collimator has a narrower penumbra, lower transmission, better mechanical stability, and a more targeted beam model.^[50]^ These advantages endow the cone plans with superior off-PTV dose fall-off.^[48]^ In particular, the significantly lower GI translates into lower OAR doses for the cone plans. For example, the brainstem, optical pathway, left lens, and bilateral hippocampal evaluation parameters were significantly lower for the cone plans (all *P* < .001). Similarly, we observed that dosimetric parameters such as *D*_mean_ and *V*_12 Gy_ in the normal brain tissues of the cone plans were superior to those of the VMAT plans, especially in *V*_8 Gy_, *V*_5 Gy_, and *V*_3 Gy_, with reductions ranging from 18.97% to 26.30%. The above results are similar to those reported by Jia-Mahasap et al,^[51]^ who found that the median OAR *D*_max_ values of the cone plans for the left optic nerve (0.6 vs 1.0 Gy, *P *< .05), right optic nerve (0.4 vs 1.4 Gy, *P* < .05), and optic chiasm (0.8 vs 1.3 Gy, *P* < .05) were all significantly lower than those of the VMAT plans.

In plans with a higher number of lesions, there was a more significant decrease in the evaluation parameters of the normal brain tissue and a significantly considerable decrease in the low-dose evaluation parameters (*V*_8 Gy_, *V*_5 Gy_, and *V*_3 Gy_) (all *P* < .05), as shown in Table 3. As the number of lesions increased, the advantage of the smaller low-dose volume of the cone plans became increasingly apparent. For example, at *V*_5 Gy_, the mean volume reduction of the cone plans for 1 to 2 lesions was 10.49 cc, while the mean volume reduction of the cone plans for 3 to 5 lesions was 37.66 cc. The above result is similar to the study of Podgorsak et al, who reported that the cone plans had a more significant advantage in normal brain tissue sparing in patients with more lesions.^[52]^ Jia-Mahasap et al^[51]^ similarly reported that, in single metastases, the cone plans had 0.7 cc less *V*_12 Gy_ than the VMAT plans, while in multiple metastases, the cone plans had 1.8 cc less *V*_12 Gy_. Therefore, to spare normal brain tissue and critical adjacent OARs, cone plans are preferentially recommended for lesions no >5.

The MUs for the cone plans did not differ significantly from the VMAT plans, suggesting that the complexity of both plans was similar – a superficially unexpected result. The underlying logic is that the cone plans are simple arc treatments with high utilization of MUs, but they are 1 treatment center per lesion.^[53]^ Therefore, when the number of lesions is small, the MUs of the cone plans are naturally less compared with those of the VMAT plans; however, when the number of lesions increases, the MUs of the cone plans will gradually catch up with or even exceed those of the VMAT plans. The slight difference in MUs between both plans in this study is that the number of lesions (1 to 2 lesions in 12 patients and 3 to 5 lesions in 11 patients) exactly hedges the advantage of high MUs utilization of the cone plans. For example, a study by Jia-Mahasap et al^[51]^ demonstrated that the MUs of the cone plans were increasingly larger than those of the VMAT plans as the number of lesions increased during SRS treatment for multiple intracranial metastases. The differences in γ-passing rates between the cone and VMAT plans under the 3 acquisition parameters were insignificant (all *P* > .05), and all met the criteria, indicating that both plans had good delivery accuracy. The above result is in keeping with the study by Čehobašić et al,^[54]^ who achieved γ-passing rates close to 100% for both plans at 1%/1 mm and 1% exclusion criteria. It has been reported^[55,56]^ that modern accelerators’ mechanical and dosimetric precision has reached a considerably high level, which is consistent with our experience in practice. The mean delivery time of the cone plans was significantly greater than that of the VMAT plans (1401.67 vs 647.07 seconds, *P *< .05), and it tended to increase linearly with the increase of the number of lesions, which caused some difficulties in the multiple-lesion patients when using cone plans. Multiple reports also support the above observation that the delivery time of the cone plans is significantly greater than that of the VMAT plans.^[21,51,57]^ This is still related to the physical characteristics of the cone collimator, which requires 1 treatment center per lesion because of its unchangeable aperture and only several discrete apertures.^[48]^ Studies have shown that prolonged treatment time decreases patient comfort and treatment accuracy.^[38,58]^ However, the prolonged treatment time seems to be the price that must be paid compared to the dosimetric advantage achieved in cone plans. To maintain the setup accuracy of the cone plans, we validated CBCT on every lesion in patients with multiple lesions when switching treatment centers.

The mean TCP values of the PTVs for both plans were above 99%, and the difference was not statistically significant, indicating that the cone and VMAT plans performed well in controlling tumor recurrence. Several studies also support these results. Pasciuti et al^[59]^ found a mean TCP of 95% for treating brain metastases using arc and fixed beams. Soltys et al^[60]^ concluded that the mean TCP of prescribed doses of 10, 11, 12, and 13 Gy in SRS for treating brain tumors was 85.0%, 88.4%, 91.2%, and 93.5%, respectively. The findings of Duan et al^[21]^ revealed that the cone plans achieved a mean TCP of 99.61% for single brain metastases, significantly better than that of the VMAT plans at 98.41% (*P* = .003). The findings of this study may be related to the high PTV dose coverage of up to 98% and 100% GTV coverage, as well as to the fact that the PTV *D*_mean_ of the cone plans was greater than that of the VMAT plans (26.47 Gy vs 25.96 Gy, *P* < .05).

The mean NTCP values of the normal brain tissues were as low as 10^−7%^ for both plans in this study. Therefore, both plans achieved reasonable complication control (low radionecrosis). Meanwhile, the mean NTCP of normal brain tissue in the cone plans was significantly lower than that in the VMAT plans (1.08 × 10^−9^ vs 3.79 × 10^−9^, *P* = .001), which may be related to the significantly lower mean GI value in the cone plans (1.79 vs 5.32, *P* < .001) and the considerably lower normal brain tissue evaluation parameters (*V*_8 Gy_, *V*_5 Gy_, and *V*_3 Gy_). Similar to our results, Tanyi et al^[61]^ found that the mean NTCP of normal brain tissue was 3.2 × 10^−3^ when the prescribed dose was 22 Gy with a 1-mm MLC margin. Duan et al^[21]^ concluded that the cone plans resulted in a mean NTCP of 1.53 × 10^−8^% of normal brain tissue for the treatment of a single brain metastasis, which was significantly superior to that of the VMAT plans of 5.84 × 10^−8^% (*P* < .05).

One limitation of the study was the relatively small number of cases, which was caused by the fact that radiosurgery treatment in our department has only been available for several years. Secondly, the radiobiological indices are derived from simplified mathematical assumptions describing tumor and normal tissue radiobiological responses, and their fixed input parameters cannot fully account for interindividual heterogeneity in normal brain radiosensitivity among patients. Further multicenter prospective enrollment will be performed with expanded sample sizes; follow-up data including long-term radiation-related adverse events and tumor recurrence will be collected to validate the calculated outputs of radiobiological models; patients with diverse primary tumor types and varying lesion sizes will be included to further validate the external generalizability of our findings.

## 5. Conclusion

In conclusion, our study demonstrates that both plans can generate high-quality SRS treatment in patients with up to 5 intracranial oligometastases. The cone plans showed greater dosimetric and radiobiological advantages than the VMAT plans; however, the delivery time of the cone plans increased significantly with an increasing number of lesions. A future direction for research is to analyze the lesion number limitations of the cone plans based on patient tolerance of delivery time, with a maximum delivery time interval.

## Author contributions

**Conceptualization:** Tingting Qin, Yangyang Huang.

**Data curation:** Jun Yang, Tingting Qin, Yangyang Huang.

**Formal analysis:** Yangyang Huang.

**Investigation:** Rui Song, Yangyang Huang.

**Methodology:** Menglin Yang, Rui Song, Yangyang Huang.

**Project administration:** Rui Song, Yibao Liu.

**Resources:** Rui Song.

**Software:** Menglin Yang, Jun Yang, Shengnan Dong, Tingting Qin.

**Supervision:** Yibao Liu.

**Visualization:** Yibao Liu.

**Writing – original draft:** Menglin Yang, Shengnan Dong, Yangyang Huang.

**Writing – review & editing:** Rui Song, Yangyang Huang, Yibao Liu.

## References

[1] Mehta MP Tsao MN Whelan TJ. The American Society for Therapeutic Radiology and Oncology (ASTRO) evidence-based review of the role of radiosurgery for brain metastases. Int J Radiat Oncol Biol Phys. 2005;63:37–46. 10.1016/j.ijrobp.2005.05.023 16111570

[2] Yamamoto M Serizawa T Shuto T. Stereotactic radiosurgery for patients with multiple brain metastases (JLGK0901): a multi-institutional prospective observational study. Lancet Oncol. 2014;15:387–95. 10.1016/S1470-2045(14)70061-0 24621620

[3] Chang WS Kim HY Chang JW Park YG Chang JH. Analysis of radiosurgical results in patients with brain metastases according to the number of brain lesions: is stereotactic radiosurgery effective for multiple brain metastases? J Neurosurg. 2010;113(Suppl):73–8. 10.3171/2010.8.GKS10994 21121789

[4] Nabors LB Portnow J Ammirati M. Central nervous system cancers, version 2.2014. Featured updates to the NCCN guidelines. J Natl Compr Canc Netw. 2014;1211:1517–23. 10.6004/jnccn.2014.0151 PMC4337873 25361798

[5] Soffietti R Kocher M Abacioglu UM. A European Organisation for Research and Treatment of Cancer phase III trial of adjuvant whole-brain radiotherapy versus observation in patients with one to three brain metastases from solid tumors after surgical resection or radiosurgery: quality-of-life results. J Clin Oncol. 2013;31:65–72. 10.1200/JCO.2011.41.0639 23213105

[6] Brown PD Jaeckle K Ballman KV. Effect of radiosurgery alone vs radiosurgery with whole brain radiation therapy on cognitive function in patients with 1 to 3 brain metastases: a randomized clinical trial. JAMA. 2016;316:401–09. 10.1001/jama.2016.9839 PMC5313044 27458945

[7] Kocher M Wittig A Piroth MD. Stereotactic radiosurgery for treatment of brain metastases. A report of the DEGRO Working Group on Stereotactic Radiotherapy. Strahlenther Onkol. 2014;1906:521–32. 10.1007/s00066-014-0648-7 24715242

[8] Nieder C Grosu AL Gaspar LE. Stereotactic radiosurgery (SRS) for brain metastases: a systematic review. Radiat Oncol. 2014;9:155. 10.1186/1748-717X-9-155 PMC4107473 25016309

[9] Fanous AA Prasad D Mathieu D Fabiano AJ. Intracranial stereotactic radiosurgery. J Neurosurg Sci. 2019;63:61–82. 10.23736/S0390-5616.17.04210-2 28945054

[10] Levivier M Gevaert T Negretti L. Gamma knife, cyberknife, tomotherapy: gadgets or useful tools? Curr Opin Neurol. 2011;24:616–25. 10.1097/WCO.0b013e32834cd4df 22071335

[11] Bedford JL Nill S Oelfke U. Dosimetric accuracy of delivering SBRT using dynamic arcs on CyberKnife. Med Phys. 2020;47:1533–44. 10.1002/mp.14090 PMC7216988 32048303

[12] Chea M Fezzani K Jacob J. Dosimetric study between a single isocenter dynamic conformal arc therapy technique and Gamma Knife radiosurgery for multiple brain metastases treatment: impact of target volume geometrical characteristics. Radiat Oncol. 2021;16:45. 10.1186/s13014-021-01766-w PMC7912819 33639959

[13] Serra M Martino FD Savino F. SBRT for localized prostate cancer: CyberKnife vs. VMAT–FFF, a dosimetric study. Life (Basel). 2022;12:711. 10.3390/life12050711 PMC9144859 35629378

[14] Yu S Xu H Sinclair A Zhang X Langner U Mak K. Dosimetric and planning efficiency comparison for lung SBRT: CyberKnife vs VMAT vs knowledge-based VMAT. Med Dosim. 2020;45:346–51. 10.1016/j.meddos.2020.04.004 32532613

[15] Balik S Chao S Neyman G. Gamma Knife and volumetric modulated arc therapy stereotactic radiosurgery plan quality and OAR sparing comparison for pituitary adenomas and vestibular schwannomas. J Radiosurg SBRT. 2018;53:237–47. PMC6018045 29988324

[16] Brezovich IA Wu X Popple RA. Stereotactic radiosurgery with MLC-defined arcs: verification of dosimetry, spatial accuracy, and end-to-end tests. J Appl Clin Med Phys. 2019;20:84–98. 10.1002/acm2.12583 PMC6522994 30977297

[17] Thomas EM Popple RA Wu X. Comparison of plan quality and delivery time between volumetric arc therapy (RapidArc) and Gamma Knife radiosurgery for multiple cranial metastases. Neurosurgery. 2014;75:409–18. 10.1227/NEU.0000000000000448 PMC4203364 24871143

[18] Liu H Andrews DW Evans JJ. Plan quality and treatment efficiency for radiosurgery to multiple brain metastases: non-coplanar RapidArc vs. Gamma Knife. Front Oncol. 2016;6:26. 10.3389/fonc.2016.00026 PMC4749694 26904504

[19] Palmiero AN Fabian D St Clair W. Management of multiple brain metastases via dual-isocenter VMAT stereotactic radiosurgery. Med Dosim. 2021;463:240–46. 10.1016/j.meddos.2021.01.002 33549397

[20] Xu MC Khattab MH Luo G. Effects of cone versus multi-leaf collimation on dosimetry and neurotoxicity in patients with small arteriovenous malformations treated by stereotactic radiosurgery. J Radiosurg SBRT. 2021;74:287–94. PMC8492055 34631230

[21] Duan Y Cao H Wu B. Dosimetric comparison, treatment efficiency estimation, and biological evaluation of popular stereotactic radiosurgery options in treating single small brain metastasis. Front Oncol. 2021;11:716152. 10.3389/fonc.2021.716152 PMC8447903 34540686

[22] Benedict SH Yenice KM Followill D. Stereotactic body radiation therapy: the report of AAPM Task Group 101. Med Phys. 2010;378:4078–101. 10.1118/1.3438081 20879569

[23] Kirkpatrick JP Wang Z Sampson JH. Defining the optimal planning target volume in image-guided stereotactic radiosurgery of brain metastases: results of a randomized trial. Int J Radiat Oncol Biol Phys. 2015;911:100–8. 10.1016/j.ijrobp.2014.09.004 25442342

[24] Crouzen JA Petoukhova AL Wiggenraad RGJ. Development and evaluation of an automated EPTN-consensus based organ at risk atlas in the brain on MRI. Radiother Oncol. 2022;173:262–8. 10.1016/j.radonc.2022.06.004 35714807

[25] Zindler JD Bruynzeel AME Eekers DBP Hurkmans CW Swinnen A Lambin P. Whole brain radiotherapy versus stereotactic radiosurgery for 4–10 brain metastases: a phase III randomised multicentre trial. BMC Cancer. 2017;17:500. 10.1186/s12885-017-3494-z PMC5526250 28743240

[26] Clark GM Popple RA Young PE Fiveash JB. Feasibility of single-isocenter volumetric modulated arc radiosurgery for treatment of multiple brain metastases. Int J Radiat Oncol Biol Phys. 2010;76:296–302. 10.1016/j.ijrobp.2009.05.029 19836151

[27] Clements M Schupp N Tattersall M Brown A Larson R. Monaco treatment planning system tools and optimization processes. Med Dosim. 2018;43:106–17. 10.1016/j.meddos.2018.02.005 29573922

[28] Gregucci F Fiorentino A Corradini S. Linac-based radiosurgery or fractionated stereotactic radiotherapy with flattening filter-free volumetric modulated arc therapy in elderly patients: a mono-institutional experience on 110 brain metastases. Strahlenther Onkol. 2019;195:218–25. 10.1007/s00066-018-1405-0 30478669

[29] Rieber J Tonndorf-Martini E Schramm O. Radiosurgery with flattening-filter-free techniques in the treatment of brain metastases: plan comparison and early clinical evaluation. Strahlenther Onkol. 2016;192:789–96. 10.1007/s00066-016-1012-x 27377261

[30] Paddick I Lippitz B. Lippitz B A simple dose gradient measurement tool to complement the conformity index. J Neurosurg. 2006;105(Suppl):194–201. 10.3171/sup.2006.105.7.194 18503356

[31] Timmerman R. A story of hypofractionation and the table on the wall. Int J Radiat Oncol Biol Phys. 2022;112:4–21. 10.1016/j.ijrobp.2021.09.027 34919882

[32] Schiff D Messersmith H Brastianos PK. Radiation therapy for brain metastases: ASCO guideline endorsement of ASTRO guideline. J Clin Oncol. 2022;4020:2271–76. 10.1200/JCO.22.00333 35561283

[33] Gondi V Pugh SL Tome WA. Preservation of memory with conformal avoidance of the hippocampal neural stem-cell compartment during whole-brain radiotherapy for brain metastases (RTOG 0933): a phase II multi-institutional trial. J Clin Oncol. 2014;32:3810–6. 10.1200/JCO.2014.57.2909 PMC4239303 25349290

[34] Korytko T Radivoyevitch T Colussi V. 12 Gy Gamma Knife radiosurgical volume is a predictor for radiation necrosis in non-AVM intracranial tumors. Int J Radiat Oncol Biol Phys. 2006;64:419–24. 10.1016/j.ijrobp.2005.07.980 16226848

[35] Blonigen BJ Steinmetz RD Levin L. Irradiated volume as a predictor of brain radionecrosis after linear accelerator stereotactic radiosurgery. Int J Radiat Oncol Biol Phys. 2010;774:996–1001. 10.1016/j.ijrobp.2009.06.006 19783374

[36] Grimm J Marks LB Jackson A Kavanagh BD Xue J Yorke E. High dose per fraction, hypofractionated treatment effects in the clinic (HyTEC): an overview. Int J Radiat Oncol Biol Phys. 2021;110:1–10. 10.1016/j.ijrobp.2020.10.039 PMC9447432 33864823

[37] Fernandez DJ Sick JT Fontenot JD. Interplay effects in highly modulated stereotactic body radiation therapy lung cases treated with volumetric modulated arc therapy. J Appl Clin Med Phys. 2020;21:58–69. 10.1002/acm2.13028 PMC7700928 33104297

[38] Tarnavski N Engelholm SA Af Rosenschold PM. Fast intra-fractional image-guidance with 6D positioning correction reduces delivery uncertainty for stereotactic radiosurgery and radiotherapy. J Radiosurg SBRT. 2016;41:15–20. PMC5658830 29296422

[39] Miften M Olch A Mihailidis D. Tolerance limits and methodologies for IMRT measurement-based verification QA: recommendations of AAPM task group no. 218. Med Phys. 2018;45:e53–83. 10.1002/mp.12810 29443390

[40] Pulliam KB Huang JY Howell RM. Comparison of 2D and 3D gamma analyses. Med Phys. 2014;41:021710. 10.1118/1.4860195 PMC3977814 24506601

[41] Okunieff P Morgan D Niemierko A Suit HD. Radiation dose-response of human tumors. Int J Radiat Oncol Biol Phys. 1995;32:1227–37. 10.1016/0360-3016(94)00475-z 7607946

[42] Gay HA Niemierko A. A free program for calculating EUD-based NTCP and TCP in external beam radiotherapy. Phys Med. 2007;23:115–25. 10.1016/j.ejmp.2007.07.001 17825595

[43] Marsh JC Godbole RH Herskovic AM Gielda BT Turian JV. Sparing of the neural stem cell compartment during whole-brain radiation therapy: a dosimetric study using helical tomotherapy. Int J Radiat Oncol Biol Phys. 2010;78:946–54. 10.1016/j.ijrobp.2009.12.012 20472348

[44] Putz F Weissmann T Oft D. FSRT vs. SRS in brain metastases-differences in local control and radiation necrosis – a volumetric study. Front Oncol. 2020;10:559193. 10.3389/fonc.2020.559193 PMC7554610 33102223

[45] Milano MT Grimm J Niemierko A. Single- and multifraction stereotactic radiosurgery dose/volume tolerances of the brain. Int J Radiat Oncol Biol Phys. 2021;110:68–86. 10.1016/j.ijrobp.2020.08.013 PMC9387178 32921513

[46] Loo M Pin Y Thierry A Clavier J-B. Single-fraction radiosurgery versus fractionated stereotactic radiotherapy in patients with brain metastases: a comparative study. Clin Exp Metastasis. 2020;37:425–34. 10.1007/s10585-020-10031-5 32185576

[47] Burman C Kutcher GJ Emami B Goitein M. Fitting of normal tissue tolerance data to an analytic function. Int J Radiat Oncol Biol Phys. 1991;21:123–35. 10.1016/0360-3016(91)90172-z 2032883

[48] Schofield A Newall M Inwood D Downes S Corde S. Commissioning of Aktina SRS cones and dosimetric validation of the RayStation photon Monte Carlo dose calculation algorithm. Phys Eng Sci Med. 2023;46:1503–18. 10.1007/s13246-023-01315-7 PMC10703943 37603132

[49] Sanvito F Telesca D Cho NS. Small pretreatment lesion size and high sphericity as favorable prognostic factors after laser interstitial thermal therapy in brain metastases. J Neurosurg. 2024;140:338–49. 10.3171/2023.5.JNS23285 PMC11787867 37542437

[50] Schmitt D Blanck O Gauer T. Technological quality requirements for stereotactic radiotherapy: expert review group consensus from the DGMP Working Group for Physics and Technology in Stereotactic Radiotherapy. Strahlenther Onkol. 2020;196:421–43. 10.1007/s00066-020-01583-2 PMC7182540 32211939

[51] Jia-Mahasap B Madla C Sripan P. Stereotactic radiosurgery for limited brain metastasis using three different techniques: helical tomotherapy, volumetric modulated arc therapy, and cone-based LINAC radiosurgery. Radiat Oncol J. 2022;40:232–41. 10.3857/roj.2022.00136 PMC9830036 36606300

[52] Yan PDY Moros PDEG. Chapter 15. In: Podgorsak EB, ed. Radiation Oncology Physics: A Handbook for Teachers And Students. Elsevier; 2006:506–48.

[53] Borzov E Nevelsky A Bar-Deroma R. Dosimetric characterization of Elekta stereotactic cones. J Appl Clin Med Phys. 2018;191:194–203. 10.1002/acm2.12242 PMC5768017 29266744

[54] Čehobašić A Paladino J Kaučić H. Comparison of 116 radiosurgery treatment plans for multi-leaf and cone collimator on a varian edge linac: are cones superior in the daily routine? Life (Basel). 2023;13:1020. 10.3390/life13041020 PMC10146576 37109549

[55] Narayanasamy G Saenz DL Defoor D Papanikolaou N Stathakis S. Dosimetric validation of Monaco treatment planning system on an Elekta VersaHD linear accelerator. J Appl Clin Med Phys. 2017;18:123–9. 10.1002/acm2.12188 PMC5689924 28944979

[56] Snyder JE Hyer DE Flynn RT Boczkowski A Wang D. The commissioning and validation of Monaco treatment planning system on an Elekta VersaHD linear accelerator. J Appl Clin Med Phys. 2019;20:184–93. 10.1002/acm2.12507 PMC6333122 30525308

[57] Hsu SM Lai YC Jeng CC Tseng C-Y. Dosimetric comparison of different treatment modalities for stereotactic radiotherapy. Radiat Oncol. 2017;12:155. 10.1186/s13014-017-0890-0 PMC5602945 28915893

[58] Lippitz B Lindquist C Paddick I Peterson D O’Neill K Beaney R. Stereotactic radiosurgery in the treatment of brain metastases: the current evidence. Cancer Treat Rev. 2014;40:48–59. 10.1016/j.ctrv.2013.05.002 23810288

[59] Pasciuti K Iaccarino G Soriani A. DVHs evaluation in brain metastases stereotactic radiotherapy treatment plans. Radiother Oncol. 2008;87:110–5. 10.1016/j.radonc.2007.12.019 18207593

[60] Soltys SG Milano MT Xue J. Stereotactic radiosurgery for vestibular schwannomas: tumor control probability analyses and recommended reporting standards. Int J Radiat Oncol Biol Phys. 2021;1101:100–11. 10.1016/j.ijrobp.2020.11.019 PMC9477217 33375955

[61] Tanyi JA Doss EJ Kato CM. Dynamic conformal arc cranial stereotactic radiosurgery: implications of multileaf collimator margin on dose-volume metrics. Br J Radiol. 2012;85:e1058–66. 10.1259/bjr/79414773 PMC3500804 23091293

